# Population-Specific Use of the Same Tool-Assisted Alarm Call between Two Wild Orangutan Populations (*Pongopygmaeus wurmbii*) Indicates Functional Arbitrariness

**DOI:** 10.1371/journal.pone.0069749

**Published:** 2013-07-05

**Authors:** Adriano R. Lameira, Madeleine E. Hardus, Kim J. J. M. Nouwen, Eva Topelberg, Roberto A. Delgado, Berry M. Spruijt, Elisabeth H. M. Sterck, Cheryl D. Knott, Serge A. Wich

**Affiliations:** 1 Animal Ecology, Utrecht University, Utrecht, The Netherlands; 2 Pongo Foundation, Oudewater, The Netherlands; 3 Institute for Biodiversity and Ecosystem Dynamics, University of Amsterdam, Amsterdam, The Netherlands; 4 Adaptation Physiology Group, Wageningen University, Wageningen, The Netherlands; 5 Human and Evolutionary Biology, University of Southern California, Los Angeles, California, United States of America; 6 Biomedical Primate Research Center, Rijswijk, The Netherlands; 7 Department of Anthropology, Boston University, Boston, Massachusetts, United States of America; 8 Research Centre in Evolutionary Anthropology and Palaeoecology, School of Natural Sciences and Psychology, Liverpool John Moores University, Liverpool, United Kingdom; University of Florence, Italy

## Abstract

Arbitrariness is an elementary feature of human language, yet seldom an object of comparative inquiry. While *arbitrary signals* for the same function are relatively frequent between animal populations across taxa, the same signal with *arbitrary functions* is rare and it remains unknown whether, in parallel with human speech, it may involve call production in animals. To investigate this question, we examined a particular orangutan alarm call – the kiss-squeak – and two variants – hand and leaf kiss-squeaks. In Tuanan (Central Kalimantan, Indonesia), the acoustic frequency of unaided kiss-squeaks is negatively related to body size. The modified variants are correlated with perceived threat and are hypothesized to increase the perceived body size of the sender, as the use of a hand or leaves lowers the kiss-squeak’s acoustic frequency. We examined the use of these variants in the same context in another orangutan population of the same sub-species and with partially similar habitat at Cabang Panti (West Kalimantan, Indonesia). Identical analyses of data from this site provided similar results for unaided kiss-squeaks but dissimilar results for hand and leaf kiss-squeaks. Unaided kiss-squeaks at Cabang Panti were emitted as commonly and showed the same relationship to body size as in Tuanan. However, at Cabang Panti, hand kiss-squeaks were extremely rare, while leaf-use neither conveyed larger body size nor was related to perceived threat. These findings indicate functional discontinuity between the two sites and therefore imply functional arbitrariness of leaf kiss-squeaks. These results show for the first time the existence of animal signals involving call production with arbitrary function. Our findings are consistent with previous studies arguing that these orangutan call variants are socially learned and reconcile the role of gestures and calls within evolutionary theories based on common ancestry for speech and music.

## Introduction

Behavioural variation that is not rooted in genetics or ecology has been described across taxa and across behavioural domains, such as the material, foraging, communicative and social domains [1]. These species have thus been interpreted as possessing rudiments of culture [1–4]. Geographic patterns in behaviour within the communicative domain, for instance, illustrate three major types of variants that may occur across animal cultures. First, a signal (with its respective function) may be present in one population but absent in another population (birds [5]:, bats [6]:, pinnipeds [7,8]:, cetaceans [9,10]:, nonhuman primates [11,12]:). Second, acoustically distinct signals with a similar function may be present in different populations (birds [13]:, cetaceans [14]:, nonhuman primates [4,15]:); such examples further imply that the signal’s *acoustic structure* is arbitrary. In other words, there is no particular relationship between the signal’s internal/external determinants (i.e. what actually triggers the signal) and its acoustic structure. In these cases the signal’s acoustic structure may take different forms within the limitations of an organism’s anatomical structures involved in call production and their respective motor control. Third, the same signal may have different functions in different populations, implying that the signal’s *function* is arbitrary. That is, there is no particular relationship between the signal’s internal/external determinants and its potential function. However, in contrast to the first two types of cultural variants, there is much less evidence for arbitrary function in animal signals and this may be restricted to great ape cultural variants [16]. Even though arbitrariness – the operational autonomy between signal production and function attribution [17] – pervades human language and underlies its unparalleled cultural richness (expressed, for instance, by the nearly 7000 world’s spoken languages [18]), it has been relatively little studied from a comparative or evolutionary perspective. The prevalence of signal arbitrariness compared to the rarity of functional arbitrariness among animal signals indicates that is distinction is relevant for understanding the nature of arbitrariness across taxa. In fact, this distinction has, indeed, been previously applied to human language by linguists and semioticians [19]. The semiotic terms “signifier” and “signified” refer to a signal itself and its function respectively, allowing for their independent analyses without assuming any fundamental relationship between them. Accordingly, this difference may offer new understanding about possible analogies/homologies across animal taxa in arbitrariness and provide new clues on language evolution.

One case of arbitrary function described in animal communication is chimpanzee leaf-clipping [16]. Chimpanzee leaf-clipping, the biting of a leaf into pieces to produce a ripping sound without eating the leaf, is used differently by chimpanzees from two different populations [16]. In one population, leaf-clipping is functionally used for courtship and in the other for play. Even though the functional differences associated with this acoustic gesture having remained essentially descriptive, they denote functional arbitrariness and have led some authors to suggest that this behaviour represents a cultural variant [2]. The first manifestation of functional arbitrariness in humans appears, however, in the infant’s prelinguistic vocalizations during first months of life [20]. Such an early emergence could be an indication of evolutionary antiquity. This observation, therefore, raises the question as to whether animal signals involving call production may also show functional arbitrariness, and, in particular, whether it occurs in our closest relatives, the great apes.

To investigate this possibility, we examined an orangutan voiceless call (or sound, sensu [21–23]), the kiss-squeak, a universal (i.e. present at all sites where orangutans have been studied) alarm call produced by a sharp intake of air through pursed lips [22]. In some (but not all) populations, the kiss-squeak is performed [4] by positioning a hand or a hand with leaves in front of or against the lips during production [23]. Correspondingly, kiss-squeaks unaided are considered innate (i.e. proper production is not dependent in auditory feedback and/or experience; sensu [24]), and hand and leaf kiss-squeaks are suggested to represent cultural variants [4,12,25]. A recent study has described how and when, and hypothesised why, orangutans at Tuanan, Central Kalimantan, use these alarm calls [23]. The positioning of a hand and leaves on their lips progressively lowers the maximum decibel frequency (Hz) of the call, but does not alter other basic acoustic characteristics, such as duration and amplitude. Orangutans produce these modified kiss-squeaks more often when confronted with perceived threats. Because the maximum frequency of unaided kiss-squeaks is negatively correlated with body size, orangutans seem to use modified kiss-squeaks to functionally deceive a potential predator by conveying a larger body size through lowering the call’s frequency with a hand or further lowering it with the use of leaves. Accordingly, at Tuanan, the distinct variants of kiss-squeaks are thought to comprise a graded three-pronged system (i.e. unaided/hand/leaves) that functionally conveys body size and relates to a perceived threat; namely, the more threatening the circumstance, the lower the kiss-squeak’s frequency.

Here, we examine potential function and correlates of kiss-squeaks with the same modifiers (hand and leaves) under the same threatening context (towards humans) in a different orangutan population from West Kalimantan – Cabang Panti – of the same sub-species ( *Pongo* *pygmaeus* *wurmbii*) and with partly overlapping habitat type (i.e. peat swamp forest). Our primary aim is to determine whether hand and leaf kiss-squeaks at Cabang Panti function to convey a larger body size and whether they relate to a perceived threat by orangutans, as in the Tuanan population. If these aspects differ between the two populations under the same context, then this call is likely to represent a signal involving call production with arbitrary function.

## Materials and Methods

### Study Site

Kiss-squeaks were recorded from wild orangutans at the Cabang Panti research station (1° 13’ S, 110° 07’ E) in the Gunung Palung National Park, West Kalimantan, Indonesia. The study area consists of seven distinct habitat types, including peat swamp forest [26]. The orangutan sub-species in this area is the same as that occurring in the Tuanan research station (2° 09’ S, 114° 26’ E), Central Kalimantan, Indonesia, composed of peat swamp forest [27]. Orangutan kiss-squeaks in Cabang Panti were recorded from February to October 2010. The stations are separated by approximately 490 Km. Research permits for entrance, research, and long-term permanence in the Cabang Panti research station were provided by the Indonesian the Ministry of Research and Technology (RISTEK), the Directorate General of Forest Protection and Nature Conservation (PHKA) and Gunung Palung National Park Bureau (BTNGP).

### Data Collection and Data Analyses

At Cabang Panti, twenty-seven identified individuals were followed during a total of 1520.6 hours using focal-animal sampling [28], comprising 5 age-sex classes [23]. Data collection did not involve direct interaction with the animals. Unaided kiss-squeaks were recorded from 21 of these individuals: immature/adolescents (n = 6; n_calls_ = 48/45/18/17/15/6), nulliparous females (n = 2; n_calls_ = 98/6), parous females (n = 7; n_calls_ = 21/16/12/10/8/6/2), unflanged males (n = 2; n_calls_ = 38/11), and flanged males (n = 4; n_calls_ = 218/65/33/21) (see Table S1). From these individuals, a sub-set of 12 individuals also emitted leaf kiss-squeaks (n_calls_ = 19/13/11/10/8/4/3/3/2/1/1/1). From all observed individuals, no audio recordings of hand kiss-squeaks were obtained. Kiss-squeaks produced by a focal orangutan and/or its associates were recorded opportunistically throughout the day with a Marantz Analogue Recorder PMD660 and a ZOOM H4next Handy Recorder with a RØDE NTG-2 directional microphone. The technique (i.e. unaided, hand- or leaf-assisted) and context (e.g. towards observers) of all kiss-squeaks was noted, using binoculars (magnification power: 10, objective diameter: 25 mm) when necessary. Whenever the technique used could not be observed directly with confidence, these recordings were not considered in the analyses. The total data analysed corresponded to 536 recordings (i.e. 30%) out of 1800 recordings. Recordings were transformed into spectrograms according to Hardus et al. [23], using Raven 1.4 (2003, Cornell Lab of Ornithology, NY, USA). To compare the acoustic structure of the different kiss-squeak techniques, the following spectrogram variables were measured: maximum frequency (Hz), maximum power (dB) and duration (s). Maximum frequency is the frequency with the highest energy emitted in a call. Maximum power is the energy of the maximum frequency (i.e. loudness). Duration represents the time period between the start and the end of the call. To control for distance to focal, volume setting, and acoustic environment during recordings, maximum power was analysed only within recording bouts [23]. Because kiss-squeaks are brief and noisy *voiceless* calls (i.e. sounds; sensu [21]), the number of measurable acoustic variables is limited. Hardus et al. [23] used the same variables, allowing a direct and accurate comparison of the calls between the Cabang Panti and Tuanan populations. All aspects of acoustic measures were kept identical to Hardus et al. [23] to allow for direct comparisons. Only kiss-squeaks emitted towards observers were considered, following Hardus et al. [23] to avoid any potential contextual biases in the analyses.

Unhabituated orangutans were those with circa 100 observation hours since 2008 (C.D. Knott, unpublished data) *and* that could not be followed during the day without showing signs of distress, as expressed by flight and/or displays. Hence, these individuals probably perceived humans as a potential threat. Individuals were considered habituated if they had more than 100 observation hours since 2008 (C.D. Knott, unpublished data) *and* could be followed without showing signs of distress. These conditions were similar to those met at Tuanan [23].

Results were qualitatively compared with those generated by data collected during 2510.0 observation hours according to the same protocol at Tuanan [23]. Within both populations, kiss-squeaks were emitted towards other orangutans and other disturbances on the ground (e.g. snakes, sun bears), in addition to humans. Non-parametric statistical tests were conducted using IBM SPSS 19 (2010, SPSS, Inc.), using a level of significance set at 0.05.

## Results

At Cabang Panti, unaided kiss-squeaks and kiss-squeaks with leaves were frequently emitted (0.47 and 0.055 kiss-squeaks/hour respectively, i.e. 714 and 84 calls over 1520.6 follow hours). However, during the same period, only 1 hand kiss-squeak was observed (i.e. < 0.001 kiss-squeaks/hour). Thus, hand kiss-squeaks were virtually absent at Cabang Panti while, at Tuanan, hand kiss-squeaks were emitted more often than leaf kiss-squeaks (i.e. 0.036 vs. 0.011 kiss-squeaks/hour, respectively [23]). Observed rates of unaided kiss-squeaks were fundamentally the same at Tuanan and Cabang Panti (i.e. 0.47 and 0.46 kiss-squeaks/hour at Cabang Panti and Tuanan, respectively). The observed rate of leaf kiss-squeaks at Cabang Panti was five-fold the rate at Tuanan (0.055 and 0.011 kiss-squeaks/hour, respectively). The rarity of hand kiss-squeaks at Cabang Panti did not allow the inclusion of this technique in subsequent analyses. These results show that, in contrast to the three-pronged functional system of kiss-squeaks at Tuanan, the Cabang Panti orangutans only use a two-pronged kiss-squeak system (i.e. unaided/leaf kiss-squeaks).

**Figure 1:**
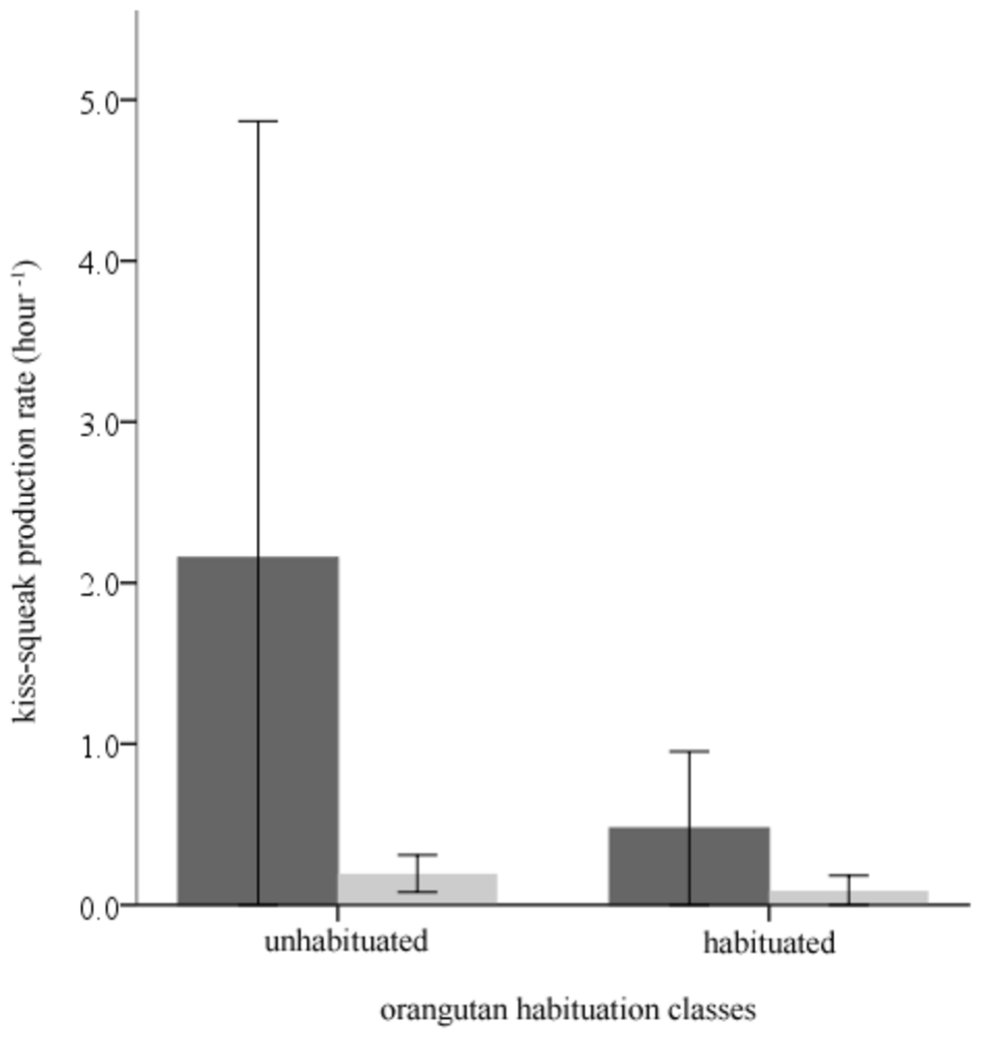
Production rates of unaided kiss-squeaks (dark grey) and leaf kiss-squeaks (bright grey) at Cabang Panti during total observation hours per individual by (A) habituated (*N* = 5) and unhabituated orangutans (*N* = 7), and by (B) habituated (*N* = 5), semi-habituated (*N* = 3) and unhabituated orangutan (*N* = 4).

**Figure 2:**
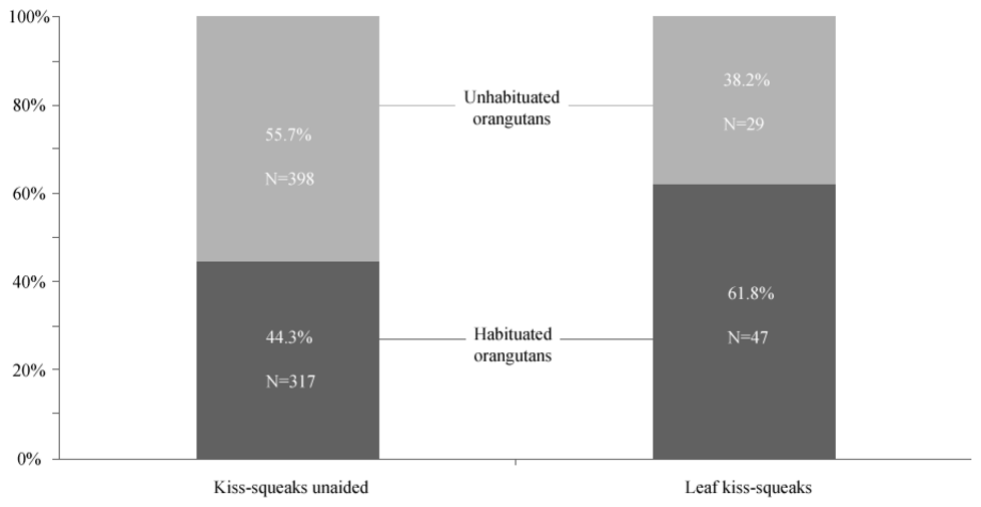
Percentage emitted by habituated and unhabituated individuals at Cabang Panti for unaided kiss-squeaks and leaf kiss-squeaks.

To assess the functional use of unaided and leaf kiss-squeaks at Cabang Panti, we compared the production rates of habituated and unhabituated orangutans towards humans. At Cabang Panti, the two kiss-squeak techniques were not produced at different rates by habituated and unhabituated individuals (Mann–Whitney *U* test: unaided and on leaves: *U* = 11.0, *N*_habituated_ = 5, *N*_unhabituated_ = 7, *P* = 0.343; Figure 1). This contrasts markedly with orangutans at Tuanan where only unhabituated individuals produced leaf kiss-squeaks towards humans [23] (Figure 2). Thus, unlike the kiss-squeak system at Tuanan, the two-pronged kiss-squeak system at Cabang Panti did not appear to relate to a perceived threat. Even though unhabituated individuals at Cabang Panti showed signs of disturbance in the presence of human observers (e.g. as expressed by flight and/or displays), this was not reflected in their use of leaf kiss-squeaks.

Error bars: +2 s.e.

**Figure 3:**
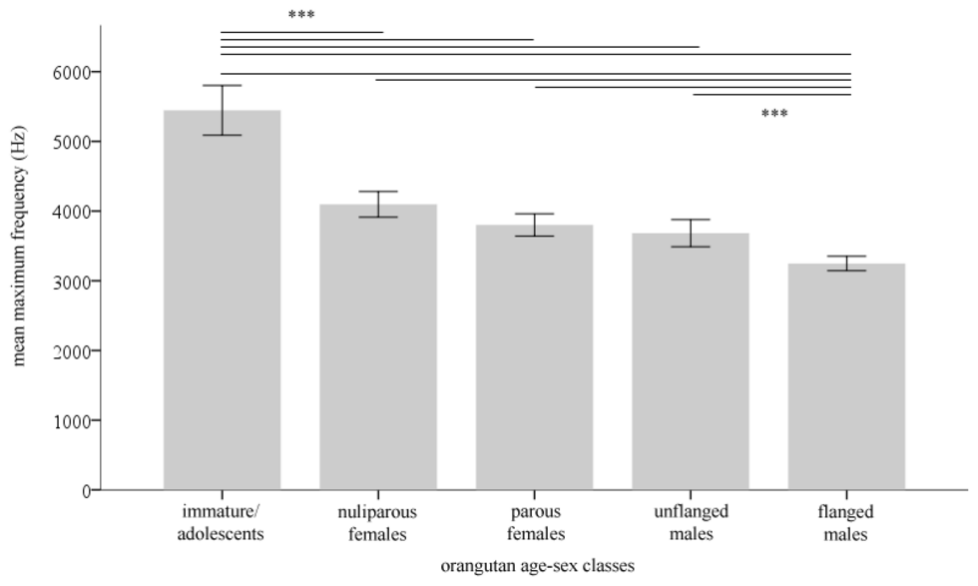
Mean maximum frequency (Hz) per orangutan age-sex class of the kiss-squeak unaided at Cabang Panti. Immature/adolescent, *N* = 6, *N*_calls_ = 149; Nulliparous female, *n* = 2, *N*_calls_ = 104; Parous female, *N* = 7, *N*_calls_ = 75; Unflanged male, *N* = 2, *N*_calls_ = 49; Flanged male, *N* = 4, *N*_calls_ = 337. Error bars: +2 s.e. *** p = 0.001.

This inter-site difference may have resulted from a lack of relationship between body size (assessed via differences between age-sex classes) and the unaided kiss-squeaks’ frequency (Hz) at Cabang Panti, in contrast to Tuanan [23]. This possibility may have precluded individuals at Cabang Panti from functionally simulating body size enlargement through tool use. However, similar to Tuanan, a negative relationship between unaided kiss-squeaks’ frequency across age-sex classes was found at Cabang Panti (Kruskal-Wallis test: *H*_4_ = 270.585, *P* < 0.001; followed by a post hoc test: *P* < 0.001 between immature/adolescents and all the other classes and between flanged males and all the other classes, Figure 3). That is, just like at Tuanan [23], the larger the body size, the lower unaided kiss-squeaks’ maximum frequency. This was not unexpected since this relationship likely reflects a physical link between the size of the source structure (i.e. lips) and the signal’s acoustic signature, similar to calls produced at the vocal folds and tract [29].

The number of observed individuals per sex-age class that emitted unaided kiss-squeaks did not differ between populations (Wilcoxon signed-ranks test: *Z* = -0.552, *N* = 5, *P* = 0.581) (see Table S1). Similarly, the number of calls recorded per sex-age class did not differ between populations (Wilcoxon signed-ranks test, *Z* = -0.944, *N* = 5, *P* = 0.345). Accordingly, it is unlikely that differences between datasets affected these results.

**Figure 4:**
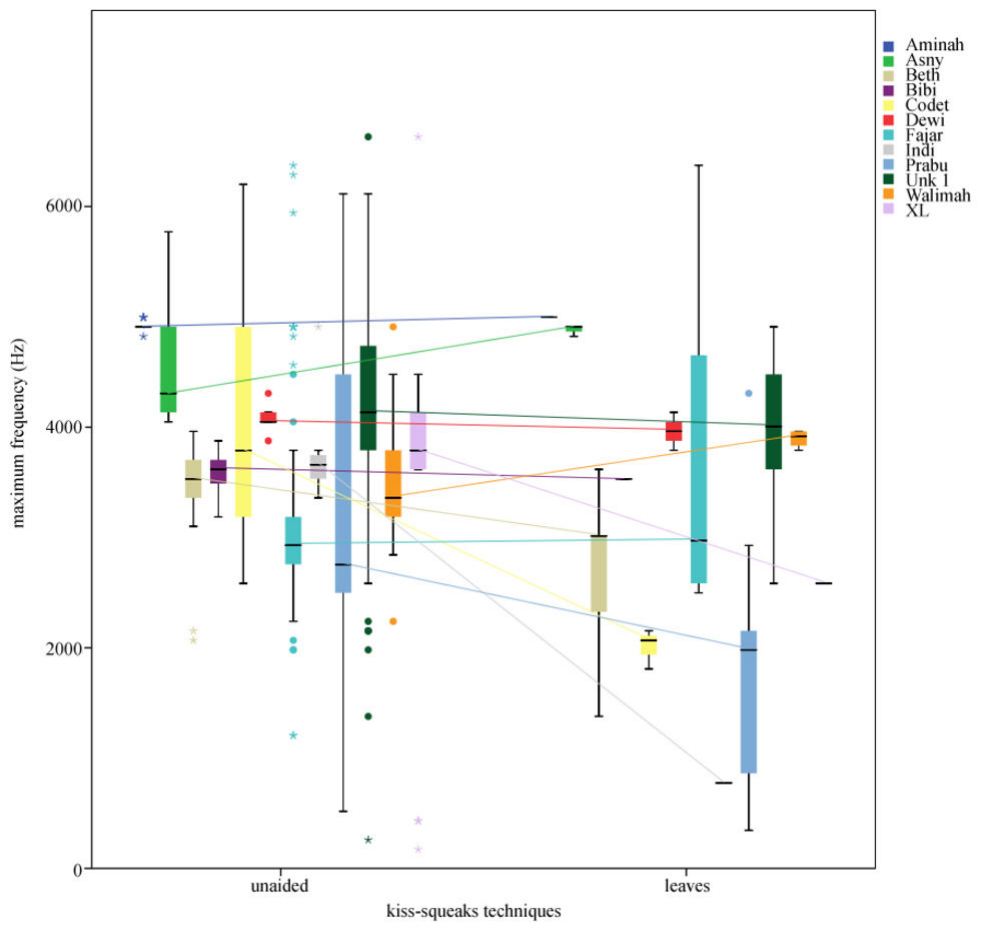
Maximum frequency (Hz) of unaided and leaf kiss-squeak for twelve orangutans at Cabang Panti.

To investigate the potential acoustic effect of leaf-use on kiss-squeak characteristics at Cabang Panti, both the acoustic characteristics of kiss-squeaks unaided and on leaves were analysed. Leaf kiss-squeaks showed a slightly lower maximum frequency than kiss-squeaks unaided produced by the twelve individuals from whom there were recordings of both variants, but this difference did not reach statistical significance (Wilcoxon signed-ranks test: *T* = -1.609, *N* = 12, *P* = 0.108; Figure 4). The difference between the median maximum frequencies of the kiss-squeak unaided and on leaves at Cabang Panti was less than 250Hz (Table 1), while at Tuanan this difference surpassed 2000Hz [23]. While positioning an item in front of the mouth during emission can lead to the decrease of a call’s frequency, sufficient proximity or contact between the tool and the lips may be crucial for larger manipulations of acoustic frequency [30]. Thus, at Cabang Panti, the manipulation of leaves was apparently not done in a manner to cause a significant acoustic effect and, contrary to Tuanan, did not functionally convey a larger body size.

**Table 1:** Quantitative acoustic differences between kiss-squeaks unaided and on leaves at Cabang Panti.

	N_ind./calls_	maximum frequency (Hz)	duration (s)	maximum power (dB)
unaided kiss-squeaks	21/714	3273 (2842.4, 4134.4)	0.493 (0.398, 0.618)	99.2 (96.4, 96.4)
leaf kiss-squeaks	12/76	3057.7 (2368.7, 4177.5)	0.562 (0.419, 0.739)	98.2 (96, 102.4)

Median values are presented with 25 and 75 percentiles between brackets.

Moreover, kiss-squeaks unaided and on leaves produced by the twelve individuals from whom there were recordings of both variants did not differ significantly in duration (Wilcoxon signed-ranks test: *T* = -0.863, *N* = 12, *P* = 0.388). Maximum power also did not differ between the two kiss-squeak techniques (Paired *t* test: *t*_26_ = 0.249, *P* = 0.805; table 1). Thus, no acoustic differences were found between kiss-squeaks unaided and on leaves within the Cabang Panti population.

## Discussion

In this study, we investigated whether functional arbitrariness may involve call production in animals, namely our closest relatives – great apes. This property emerges in human pre-linguistic vocalizations in the first months of life [20], and underlies the subsequent acquisition of any spoken language by any individual [18,19]. However, functional arbitrariness has never been approached from a comparative or evolutionary standpoint. Here, we examined the use and potential function of three orangutan call variants emitted towards humans at Cabang Panti, in order to compare them with another population, Tuanan, where these calls’ use and function in same context are known [23]. When the use and potential function of one of these variants differ between populations, this will be an indication of functional arbitrariness.

In sum, our results show that, at Cabang Panti, leaf kiss-squeaks towards humans did not function to convey body size enlargement and its use did not relate to perceived threat, despite being produced relatively frequently. This contrasts with their use at Tuanan where, in the same context, orangutan leaf kiss-squeaks functionally convey a larger body size and relate to a perceived threat [23]. Also, in contrast with Tuanan, hand kiss-squeaks were absent at Cabang Panti and habituated individuals produced leaf kiss-squeaks towards observers.

There were several potential factors that could have influenced these results. A difference between the two datasets could have biased the comparison between populations, but our analyses demonstrate that this was not the case. Another factor that could have influenced our results are habitat differences, which have been reported to influence the acoustic characteristics of calls over distance [31]. However, habitat differences between the two populations studied here are unlikely to have affected our results for at least five reasons. First, individuals at Cabang Panti used leaves indiscriminately of plant species in a similar fashion to orangutan in Tuanan [23], who simply used any leaves within reach at the moment of disturbance. Second, the possibility of differences in the type of leaves randomly picked between sites is further unlikely, as the sites are composed by overlapping habitat types. Third, even though differences in leaf kiss-squeaks’ maximum frequency were found between sites, this parameter is known to remain stable over close- and middle-range distances [31]. That is, a distance range within which call recordings were collected at both sites. Fourth, habitat acoustics exert little selective pressure over primate calls [32]. Fifth, habitat-driven differences in the acoustic structure of calls preserve call functionality [33]. Habitat differences do not drive functional divergence.

Human factors are also unlikely to have affected the results. Human observers at both sites collected data according to the same standardized methods [34]. Thus, we do not suspect that observers behaved in any different way between sites that could have resulted in orangutans also behaving differently. We are also unaware of any possible means by which observers’ behavior could produce the specific results obtained. This similarly applies to potential differences in orangutans’ habituation level between sites.

Due to the close genetic relatedness between the two orangutan populations, involving the same sub-species and with a recent mean coalescence date of much less than 176ka [35], it is unlikely that genetic differentiation is the source of the differences found in this study. In addition, to our knowledge, there is no known genetic process which codes and influences a particular behaviour differently in the same context (thus, probably under the same affective state (sensu[36]) of the individual) with or without tools, and that can be expressed differently within a particular sub-species. This would seem particularly improbable in the specific case of alarm calls which are expected to be under high evolutive inertia due to their importance for individual survivorship (e.g. [37]). Indeed, the geographic patterning of presence or absence of leaf kiss-squeaks is neither sufficiently explained by genetic differentiation between populations nor environmental differences [3]. This observed variation concurs with evidence demonstrating that genetic differentiation does not sufficiently explain the geographic pattern of other orangutan calls [15]. Conversely, orangutans have been empirically shown capable of observational learning [38]. Moreover, proficient use of tools in wild non-human primates is assumed to depend on practice (e.g. [39,40]). Altogether, the results from this study concur with the literature suggesting that leaf kiss-squeaks may be socially learned within each population and that they represent cultural variants [3,4,23]. Future controlled experiments in captivity may assess the role of social learning in more detail.

As far as we are aware, this is the first study to directly demonstrate that an animal signal involving call production does not present a continuous and uniform function across populations. Although, in a broad sense, orangutan leaf kiss-squeaks constitute an alarm call at both Tuanan and Cabang Panti, leaf kiss-squeaks at Cabang Panti do not satisfy the predictions for the specific deceptive function proposed for Tuanan [23]. This demonstrates that a particular function attributed to a signal may be different across communities, implying arbitrariness in its relationship to the signal. Thus, although the identification of the specific function of leaf kiss-squeaks at Cabang Panti remains uncertain, it is the absence of Tuanan’s function at Cabang Panti that expresses the arbitrary nature of whichever functions are potentially attributed to this signal. Future experiments using playbacks could further investigate the function of leaf kiss-squeaks at Cabang Panti. However, this may prove challenging since the acoustic features of leaf kiss-squeaks at this site provide few clues. In fact, leaves seem to serve no apparent *acoustic* function as a tool, and thus, the signal may have been maintained within the population but not its function. On the other hand, the function of the leaves could have transferred between domains to become a visual enhancement to the kiss-squeak acoustics, as it may occur in other species [41]. This would explain the absence of hand kiss-squeaks in the Cabang Panti population, since these serve no conspicuous visual enhancement.

The differences we report here between these orangutan populations differ from other types of variation described in animal signals in at least three fundamental ways. Leaf kiss-squeak differences are observed in the absence of any contextual variation (e.g. [42,43]), they do not constitute one of the species’ innate/universal calls, and they involve tool use. Interestingly, the two examples of great ape signals with arbitrary function – chimpanzee leaf-clipping [16] and orangutan leaf kiss-squeaks (this study) – are directly connected with and dependent on tools. This may suggest that the use of a manipulated external element (i.e. tools), executed in synchrony with a communicative signal, may facilitate an operative uncoupling at the neurocognitive level between a signal’s internal/external determinants and its attributed function [44]. This finding is relevant to the theory of speech evolution, since gestural and acoustic models of language evolution are commonly seen as mutually exclusive [44]. Our results indicate that, although non-human primate calls are commonly considered affect-based (sensu [36]) and innate (sensu [24]), namely alarm calls due to their importance for the individual’s survival, advanced communicative features, such as arbitrariness, may be brought about into the call domain when gestures and calls are synchronous and conjoint [44]. This is the case even when calls are voiceless, as is the case of orangutan kiss-squeaks [30]. These results are in concordance with “musilanguage” models of human language and speech evolution [45] in that they conjure a multicomponent [41] common precursor to human language and music, but also suggest that, within this evolutionary model, the role of tools as “musical instruments” may have been much more relevant than previously assumed.

Evidence of arbitrary signals [15] and signals with arbitrary function (this study) in great apes suggests that primatologists, evolutionary anthropologists, acoustic biologists, and scholars interested in comparative biology may benefit from the use of the linguistic and semiotic terms such as “signifier” and “signified” to refer to a signal and its functional use, respectively. When discussing and investigating arbitrariness in the context of comparative biology, it is relevant to specify whether signal or functional arbitrariness is addressed. The relative abundance of examples of the former versus the rarity of examples for the latter in animal communication (see Introduction) suggests that these two features may be underlined by different cognitive mechanisms. Their convergence in great apes suggests that our closest ape relatives may have already been under positive selection towards increased motor control over signal production. In particular, signals involving call production with arbitrary function may date back to the homininae-ponginae evolutionary split (i.e. 9-13 MYA [46]) and may have paved the way towards the use of words with arbitrary meaning in human speech.

## Supporting Information

Table S1 Number of subjects per age-sex class and habituation level. (DOCX)
