# Correction: Population-Specific Use of the Same Tool-Assisted Alarm Call between Two Wild Orangutan Populations (*Pongo pygmaeus wurmbii*) Indicates Functional Arbitrariness

**DOI:** 10.1371/annotation/693d4518-22f0-4f44-82e1-8d7bc7f5f0fd

**Published:** 2013-08-22

**Authors:** Adriano R. Lameira, Madeleine E. Hardus, Kim J. J. M. Nouwen, Eva Topelberg, Roberto A. Delgado, Berry M. Spruijt, Elisabeth H. M. Sterck, Cheryl D. Knott, Serge A. Wich

The correct version of the title is: Population-Specific Use of the Same Tool-Assisted Alarm Call between Two Wild Orangutan Populations (*Pongo pygmaeus wurmbii*) Indicates Functional Arbitrariness

The correct citation is: Lameira AR, Hardus ME, Nouwen KJJM, Topelberg E, Delgado RA, et al. (2013) Population-Specific Use of the Same Tool-Assisted Alarm Call between Two Wild Orangutan Populations (*Pongo pygmaeus wurmbii*) Indicates Functional Arbitrariness. PLoS ONE 8(7): e69749. doi:10.1371/journal.pone.0069749 

