# Supplementary material for: Population-Specific Use of the Same Tool-Assisted Alarm Call between Two Wild Orangutan Populations (Pongopygmaeus wurmbii) Indicates Functional Arbitrariness
Source: PLoS One. 2013 Jul 5;8(7):e69749. doi: 10.1371/journal.pone.0069749 (PMC3702587; doi:10.1371/journal.pone.0069749)
Supplement: Table S1 — (DOCX) [file pone.0069749.s001.docx]

Table S1. Number of subjects per age-sex class and habituation level

|  |  | immature/  adolescents | nulliparous  females | parous  females | unflanged  males | flanged  males |
| --- | --- | --- | --- | --- | --- | --- |
| Cabang Panti | habituated | 4 | 1 | 3 | 1 | 2 |
|  | unhabituated | 2 | 1 | 4 | 1 | 2 |
| Tuanan | habituated | 3 | 1 | 4 | 1 | 1 |
|  | unhabituated | 0 | 1 | 1 | 8 | 6 |
